# A Review of Flipped Classroom and Cooperative Learning Method Within the Context of Vygotsky Theory

**DOI:** 10.3389/fpsyg.2020.01157

**Published:** 2020-06-03

**Authors:** Deniz Gökçe Erbil

**Affiliations:** Department of Primary Education, Faculty of Education, Karamanoglu Mehmetbey University, Karaman, Turkey

**Keywords:** cooperative learning, flipped classroom, Vygotskian < Theoretical perspectives, zone of proximal development (ZPD or ZOPED), social interdependence

## Abstract

In the flipped classroom method, which is accepted as one of the blended learning approaches, the traditional teaching process takes place outside of the classroom through videos. Activities, projects, and homework related to upper-level cognitive field steps are carried out during classroom time. Research and interest in the flipped classroom are increasing steadily. Employing a cooperative learning method is suggested for using class time in the flipped classroom method. However, there has not been sufficient research on the implemented results of those suggestions. Moreover, there is no clear roadmap on how to incorporate cooperative learning methods into the flipped classroom. This research reviews theoretical infrastructures of flipped classroom and cooperative learning methods according to the Vygotsky theory and makes various suggestions for implementation and implementers.

## Introduction

Constructivism has a strong impact on the modern learning-teaching process as a dominant education philosophy. The learning approaches and teaching methods based on constructivism are influenced by the theories of Piaget and Vygotsky (Tzuo, 2007). In active learning methods developed based on constructivist theories, the student plays the role of the constructor of information and takes an active role (Piaget, 1968; Vygotsky, 1978). Active learning is defined as the moment when the teacher stops teaching a lesson and students work on a question or task provided to them to understand a subject (Andrews et al., 2011). In another definition, active learning is defined as any teaching method that engages the student into learning process. For example, cooperative learning, problem-based learning, and project-based learning are accepted as active learning methods and have been implemented for a long time. Based on this definition, students must carry out meaningful learning activities and think about what they are doing in an active learning process (Bonwell and Eison, 1991).

The flipped classroom method, which argues that students must be active during class time and must structure information within themselves and their own process, is one of the active learning methods (Berrett, 2012; Milman, 2012; Strayer, 2012; Munir et al., 2018). Flipped classroom method can be defined as carrying the traditional teaching method out of the classroom through online videos. The transfer of information is carried out via videos watched by students out of the classroom. In this direction, active learning methods are used instead of traditional ones during the class time, and the students are able to deepen their learning (Foldnes, 2016). In the flipped classroom, the process of transferring information in traditional classrooms goes outside the classrooms through computer technologies and the Internet, and the “information transfer” is carried out by interactive activities within an active learning environment (Berrett, 2012). Flipped classroom is where the activities carried out in the classroom environment in traditional learning approach (lecturing, teaching of concepts) are carried out of the classroom with the help of technological means, and the activities that are conducted outside of the classroom environment in traditional approach (homework, projects, upper-level activities) are carried out within the classroom environment. Bishop and Verleger (2013) based their reasoning on including the flipped classroom in active learning methods on the student-centered learning theories of Piaget (1968) and Vygotsky (1978). According to both Piaget and Vygotsky, the outer world and the interactions carried out with the outer world play an important role in an individual's development. While Piaget refers to the concept of cognitive conflict that occurs as a result of peer interactions, Vygotsky explained the learning that occurs as a result of interactions with individuals that are more advanced than the first with the zone of proximal development (ZPD) concept (Tudge and Winterhoff, 1993). Both theoreticians highlight the fact that the learning process depends on interactions with others and the importance of the reflection of this interaction process to an individual's inner world.

The cooperative learning method, which was defined as utilization of small groups for educational purposes (Johnson et al., 2007), is a teaching method developed for over 40 years, and the effects of which on various areas such as student success, attitude, and motivational levels are proven by research (Johnson et al., 2000; Hattie, 2009; Kyndt et al., 2013; Kocabaş et al., 2015; Dirlikli et al., 2016; Erbil and Kocabaş, 2018). Cooperative learning is a learning approach in which students work in small, independent groups for common educational goals, and their work is evaluated both individually and as a group. According to Johnson and Johnson (2009), the social interdependence theory brought forward by Lewin and Deutsch is the foundation of the cooperative learning method. Social interdependence theory posits that individuals are affected by their own acts or those of other individuals (Johnson and Johnson, 1989). This theory was improved by Johnson and Johnson (2009) and designated the cooperative learning method. Cooperative learning study must have five basic characteristics: positive interdependence, individual accountability, face-to-face promotive interaction, interpersonal and small group skills, and group processing (Johnson et al., 2000, 2007; Sharan, 2015). These five basic elements are what separate the cooperative learning method from other group work methods and may also be defined as the core principles providing the power of the method.

During class time in the flipped classroom, any active learning method can be used. Discussion (Wei et al., 2020), problem solving (Khanova et al., 2015), brainstorming (Kong, 2014), concept mapping (Porcaro et al., 2016), students' presentations (Wang et al., 2019), and gaming (Jo et al., 2018) were used as in-class activities by researchers. However, using a cooperative learning method in flipped classrooms is especially suggested by some researchers (Bergmann and Sams, 2012; Tucker, 2012; McLaughlin et al., 2013; Flynn, 2015; Long et al., 2017; Spector and Ball, 2017). In a flipped classroom environment, the lesson content is transferred to the online environment via videos. Students come to class after having watched these videos. Learning activities suitable for upper-level cognitive field achievements must be carried out in the classroom environment. At this point, the cooperative learning method is one of the most suitable teaching methods for flipped classroom environment (Bishop and Verleger, 2013; Betihavas et al., 2016; Lai and Hwang, 2016). There are few research studies in which the cooperative learning method is applied in a flipped classroom environment (Foldnes, 2016). Utilization of the cooperative learning method in a flipped classroom environment is at a development stage, and there are no clear data regarding its results (Munir et al., 2018). However, the existing research has concluded that utilizing cooperative learning methods in a flipped classroom environment has a positive impact on students' academic success levels (Chen et al., 2015; Foldnes, 2016; Guo et al., 2018; Munir et al., 2018; Zhang, 2018).

## The Goal of the Research

This research will review the flipped classroom and cooperative learning method in aspect of Vygotsky's learning theories. As a result of this review, common points of cooperative learning and flipped classroom will be established. This research will then make some suggestions on how the flipped classroom will be integrated into the cooperative learning method. It is expected that the outcomes will benefit researchers, the implementation process, and the implementers.

## Flipped Classroom and Vygotsky Theory

The flipped classroom environment is one in which active learning methods are used (Betihavas et al., 2016). Its conceptual foundations are based on simply not teaching the lessons in a classroom environment and on student-centered learning theories (Piaget, 1968; Vygotsky, 1978). In a flipped classroom, the information—the part given face-to-face in a traditional approach—is taken out of the classroom in an active and cooperative way (Strayer, 2012; Chen et al., 2015; Betihavas et al., 2016; Foldnes, 2016; Lai and Hwang, 2016; Zhang, 2018). Students prepare for the lesson using the resources used in a traditional lesson. When they come to the classroom environment, they share the information they acquired with their classmates. The flipped classroom is basically a learning model that aims to eliminate the traditional learning approach in which students are generally passive and which is based only on transfer of information. In the flipped classroom, students are active during the lesson and have the role of structuring the information (Munir et al., 2018). This role is carried out by applying activities suitable for upper-level cognitive field achievements in classroom environment in school (Bergmann and Sams, 2012; Sarawagi, 2013). Because the traditional teaching approach is flipped, in the classroom environment (or small group works), which is a large learning group, the individual has the opportunity to experience learning with their classmates and also internalizing these experiences individually. Looking from the aspect of these assumptions, flipped classroom is a teaching model that is suitable for active learning approach.

In a flipped classroom, unless class time is enhanced with active learning methods, it can depart from being an approach that is based on constructivism. In other words, the effectiveness of a flipped classroom depends on using class time for strong and successful activities. Solely taking the lesson content out of the classroom via videos does not guarantee effectiveness of learning. Moreover, it is imperative that class time be structured based on active learning methods and activities.

While Bishop and Verleger (2013) are the first researchers to link flipped classroom and Vygotsky theory, some research carried out afterward has tried to strengthen this link. According to Maciejewski (2016), in a flipped classroom environment, there is more available time to be utilized within the classroom. This time can be structured so that students are able to communicate and interact with each other more in the classroom. Students can also work within the group during this time and interact with each other for problem-solving exercises. Hao (2016) has also approached flipped classroom over Vygotsky's (1978) point of view, and in this theory, the interaction of individuals is quite important. Therefore, in his research, he provided the students opportunity to work in groups to enable them to help each other more.

Vygotsky (1978) emphasizes two main points in the learning process. The first is culture, and the second is language.

### Flipped Classroom as a Means of Cultural Transmission

The most important point that emerges when we approach the flipped classroom using the Vygotsky theory is that the flipped classroom provides a suitable environment for transmission of culture. The flipped classroom does this in two ways: (1) videos prepared as lesson content and (2) class time in which active learning methods are used.

### Vygotsky, Language, and Flipped Classroom

According to Vygotsky (1962, 1987), language is improved with social interactions carried out for communication purposes and has two roles that are critical for cognitive development. The first role of language is that it is the means of transmission of knowledge of adults to the child, and the second is that language is single-handedly a very powerful means that provides for the child's intellectual harmony. This section will focus on the first role of language, because the second role pertains more to results of internal and external speech carried out by the individual (Vygotsky, 1987). As a result of the individual's social interactions, their talks with others, their talks with themselves privately, and their internal talks are more related to cooperative learning, so they will be mentioned in that section.

In a flipped classroom, the teacher prepares lesson content as a video and puts it on the Internet. The students watch those videos in extracurricular time, and they gain lesson content. Vygotsky (1962) emphasizes the importance of language in transmission of culture. The rules of society value judgments; in short, anything regarding social structure is structured in spoken language and transmitted to the child via language. Therefore, in a flipped classroom, lesson content, in other words, teaching programs prepared by the institutions, the values, rules, restrictions, and so on, to be transmitted to students, is transmitted via the videos. As a more knowledgeable person, the teacher continues this role within the classroom and also carries this role to out-of-school time. A more knowledgeable person has an important role in a child's learning, in Vygotsky theory (Vygotsky, 1978). In the traditional teaching-learning process, the teacher (therefore managers) cannot interfere in extracurricular time. At maximum, they present textbooks, workbooks, and Internet resources to students, and students process information by themselves in homework, activities, and project tasks. However, in a flipped classroom, students watch videos in extracurricular time, and at this point, language plays an important role as a culture transmission tool. Furthermore, the homework, activity, and project process in which the student works on his/her own and leans on internal speak more is carried to the school environment. From this point of view, social structures and means direct the student both in the school environment and at home in a flipped classroom. The transmission of culture continues incessantly in and outside of school.

*Proposition 1:* The flipped classroom utilizes the videos in extracurricular time as a means of culture transmission. In social aspect, social structures, tools, and values are transmitted to students in extracurricular time.

### A More Knowledgeable Person, Zone of Proximal Development, and Flipped Classroom

In a flipped classroom, it is important to engage active learning methods in the class time and use them (Bishop and Verleger, 2013; Roehl et al., 2013; Gopalan et al., 2018). Active learning is an umbrella term that consists of the teaching methods focused on participation of students in the learning process and their loyalty (Prince, 2004). However, in active learning process, students do not only learn on their own, but the teacher is also a guide in active learning, and the student's peers are also included in each other's learning process.

Vygotsky (1978) also argues that cognitive development is carried out via social interactions. He states that the learning process will continue more effectively as a result of interactions students engage in with peers that are more knowledgeable or adults (e.g., teacher, family). His principle of ZPD is based on the difference between things an individual can do on his/her own independently and without any help and those he/she can do with the help or encouragement of a peer or an adult (a more knowledgeable person). The individual learns more effectively as a result of an interaction with more knowledgeable peers or adults. Moreover, it is based on the view that the individual can do things with the help of others without any help. In a flipped classroom, in extracurricular time, the individual watches the lesson content videos prepared by an adult who is more knowledgeable (teacher). If the active learning methods that encourage the individual to gather around with their peers and provide such an environment are used in class time, the individual's learning process will be positively impacted (we hereby refer to cooperative learning). When the individual carried out the upper-level cognitive studies, activities, and homework together with their peers, especially the students with lower levels of success will interact with peers who are more knowledgeable than they are and therefore will benefit from them within the context of ZPD.

Thus, if the flipped classroom's achievement is aimed according to Vygotsky theory, class time must be carried out with teaching methods that are based on active learning methods in which the students will work together as a group and benefit optimally from each other in terms of information. At this point, cooperative learning method is one of the most suitable teaching methods that can be included in the flipped classroom, and this result is supported by the research in literature.

*Proposition 2:* If we aim to benefit from the flipped classroom method to the maximum level according to Vygotsky theory, the active learning approaches that have students interact with their peers and that support group work must be preferred.

## Cooperative Learning Method and the Vygotsky Theory

Even though they lived in the same period, Lewin and Vygotsky conducted research on similar issues completely independent of and unbeknown to each other. Vygotsky also conducted important research that empowers the theoretical framework of cooperative learning. In 1970s, while several research projects on cooperative learning had already been made in Western literature, the works of Vygotsky were translated into English as late as 1978. Vygotsky entered the educational sciences literature of Europe and the United States after this date. However, according to the radical impact of constructivism on individual learning and internalization of information on the Western society and culture, the fact that Vygotsky talks about “constructivism,” even under other names, in his works written in 1920s, has created a great impact. In his book entitled *Mind in Society*, translated into English in 1978, Vygotsky approached humans as beings in interaction with other individuals as opposed to individual, lonely beings while explaining the cognitive development of humans. According to him, cognitive development is the skill of the individual to learn how to use suitable social tools (e.g., car, mobile phone, money, etc.) and cultural signs (writing, language, numbers) via their peers and teachers that provide for cultural socialization of the individual and in social interaction. He argues that a child learns to carry out simple cognitive activities (such as basic perceptions, attention without awareness, etc.) first. Then they gain the upper-level skills by interacting with peers and teachers in a social environment. These upper-level skills are skills such as language, mind, problem-solving, and moral reasoning. Another concept Vygotsky worked on is internalization. He defines internalization as the individual experiencing a thought, behavior, or attitude in a social environment for the first time and making this experience cognitively functional. He argues that the social interactions and cultural signs are important in an individual's learning process. Individuals must get into social interaction with peers that are more competent and knowledgeable or their family–teachers in order to improve their own learning or learn a new subject. Using cultural signs for this interaction is very important (Vygotsky, 1978).

Zone of proximal development is one of Vygotsky's most important theories. Zone of proximal development is defined as “the difference between the developmental level of the individual at that moment defined by their independent problem-solving skills and their potential developmental level to be reached as a result of adult guidance or their collaboration with their more advanced peers” (Vygotsky, 1978).

### Cooperative Learning and Zone of Proximal Development

This section of the research will review the relation between ZPD and cooperative learning. In this review, Moll's (1990) three basic keys to understanding the Vygotsky theory will be evaluated in aspect of evaluation of positive dependency, face-to-face supportive interaction, assessment of group process, individual accountability, and interpersonal and small group skills, which are the basic characteristics of cooperative learning (Johnson et al., 2000, 2007).

Moll (1990) stated that Vygotsky's ZPD theory depends on the understanding of three basic issues. These are holistic use of authentic activities, the need for social interaction, and the individual's process of change.

#### Holistic Use of Authentic Activities

According to Vygotsky, the process of working, learning, and teaching must be carried out holistically by teachers and must be realized via authentic activities. Authentic activities must be based on real-life situations and must be meaningful to the students. Students must also feel the need to learn that subject (Vygotsky, 1978).

#### The Need for Social Interaction

According to Vygotsky, students learn via the social interactions they engage in with their more competent peers or their teachers. Within this interaction, students who have newly acquired information from their more competent peers or teachers learn it directly through primary experience, by doing and living, and internalize it (Vygotsky, 1978). This process is seen by Doolittle (1997) as the most important proof of social dependency. Here, ZPD is not only single-sided according to students, peers, or teachers, but it is also interactive. Social interaction is a condition in ZPD, and social dependency is a prerequisite between individuals in ZPD process.

#### The Individual's Process of Change

Vygotsky (1987) argued that the objective of cognitive development is the change of the individual. According to him, ZPD is an ongoing change. As individuals learn and improve themselves, the interactions they go into with other individuals lead to culturally necessary changes in their behavior.

#### Positive Dependency

According to Doolittle (1997), there is a very important relation between Vygotsky's views and positive dependency. Each individual is dependent on the other individuals in the society with regard to presentation of resources that will be beneficial for their cognitive development. The society is, in turn, responsible for enculturation of the individual, that is, transmission of the existing culture (Valsiner, 1988).

#### Face-to-Face Interaction

The correspondence of face-to-face interaction in Vygotsky theory is social mediation and enculturation. Social mediation is the individual gaining information and skills via social interactions. Transmission of society's cultural signs to the individual and utilization of these signs by the individual in the learning process is defined as enculturation. Enculturation is related to “what” is learned, whereas social mediation is more related to “how” it is learned (Doolittle, 1997).

#### Individual Accountability

In Vygotsky theory, individuals are responsible for developing their own zones of proximal development. Each individual must learn within the lesson process and advance their own learning. For example, in group work, the individual must be able to repeat a skill they could do with the group yesterday by themselves and without help today (Doolittle, 1997).

#### Interpersonal and Small Group Skills

In both cooperative learning and Vygotsky theory, interpersonal and small group skills are used. In Vygotsky theory, individuals use cultural signs (writing, pictures) in social interactions. Cultural signs are important tools in the social mediation and enculturation process (Vygotsky, 1978; Doolittle, 1997).

#### Assessment of Group Process

According to Doolittle (1997), assessment of the group process occupies a major place within ZPD because, in the ZPD process, both their peers and their teacher are responsible for the individual's learning. Moreover, a learning goal that is even lower than the lower limit of ZPD level will be boring to the individual. On the other hand, a learning goal that is beyond the individual's ZPD level will be found very difficult by the individual and probably will not be achieved. In both possibilities, the individual will not be able to acquire the desired behavior. Therefore, it is very important to keep the learning goal within ZPD.

The cooperative learning method was not developed by Vygotsky. However, his approach (1978) is similar to the roots of the cooperative learning method. The five basic characteristics of cooperative learning (positive dependency, face-to-face supportive interaction, individual accountability, interpersonal and small group skills, and assessment of group process) are closely related to the three basic issues argued by Vygotsky, namely, holistic use of authentic activities, need for social interactions, and the individual's process of change.

*Proposition 3:* One of the major learning approaches that might carry Vygotsky's (1978) ZPD approach in teaching–learning process is the cooperative learning method.

### Cooperative Learning Method and Flipped Classroom

According to Tzuo (2007), student-centered learning theories, and therefore the teaching methods that arise out of those theories, are heavily based on the cognitive constructivism theory of Piaget and social constructivism theory of Vygotsky. Both theories state that individuals structure information within themselves. However, each theory explains how this structuring occurs differently.

Piaget's point of view is that structuring of information majorly depends on the previous experience of the individual; learning occurs as a result of the individual's interactions with the environment (Lourenço and Machado, 1996; Tzuo, 2007). Vygotsky emphasized the importance of interaction between individuals in construction of information. He also stated that cultural-historical and personal factors are the basic elements of human development (Tudge and Scrimsher, 2003). Zone of proximal development is one of the most important concepts in Vygotsky theory. According to Puntambekar and Hubscher (2005), ZPD refers to the level or developmental rank, which students can achieve under suitable educational conditions.

Dockett and Perry (1996) state that the main difference between Piaget and Vygotsky is related to the direction of the impact of social interaction. In Piaget's theory, information is formed by the individual through experience. This information is tested and edited within a social interaction process, whereas in Vygotsky theory, information is structured in the social structures within the cultural heritage in which the learning process does not occur and in the social relations (Cole and Wertsch, 1996; Dockett and Perry, 1996). Schunk (2012) says that, in Vygotsky theory, the methods of interaction with the persons, objects, and institutions in students' world change their thoughts.

Another difference between Piaget and Vygotsky occurs in explanation of developmental phases. While Piaget explains developmental phases steadily, Vygotsky makes a more flexible and fluid explanation (Blake and Pope, 2008). The cognitive development phases presented by Piaget have continuity and transformation and are viable for each person, although differing according to maturity, experience, and cultural conditions (Ojose, 2008). Vygotsky utilizes ZPD to explain developmental phases and emphasizes that social impacts such as collaboration and structural ladder in learning are the most important concepts for explaining development (Dockett and Perry, 1996; Vianna and Stetsenko, 2006).

In the flipped classroom method, active learning methods are used in class time (Bishop and Verleger, 2013; Roehl et al., 2013; Gopalan et al., 2018). Active learning is an umbrella term that encompasses the teaching methods focused on participation of students in the learning process and their engagement (Prince, 2004). Therefore, we can conclude that there is an active learning method in a flipped classroom and that it is shapes as a teaching method based on constructivist theories.

The cooperative learning method, dating back to a much older time and an older history of literature than the flipped classroom, is among the active learning methods (Johnson et al., 1995; Keyser, 2000). Therefore, the cooperative learning method is based on constructivist theories, as well.

During class time in the flipped classroom, any active learning method can be used. However, using a cooperative learning method in flipped classrooms is especially suggested by some researchers (Bergmann and Sams, 2012; Tucker, 2012; McLaughlin et al., 2013; Flynn, 2015; Long et al., 2017; Spector and Ball, 2017).

In a flipped classroom environment, lesson content is transferred to the online environment via videos. Students come to class having watched these videos. The learning activities suitable for upper-level cognitive field achievements must be carried out in classroom environment. At this point, the cooperative learning method is one of the most suitable teaching methods for flipped classroom environment (Bishop and Verleger, 2013; Betihavas et al., 2016; Lai and Hwang, 2016).

The research shows that the flipped classroom method has a positive impact as an active learning method on various attitudes and behaviors such as student success (Bhagat et al., 2016), motivation (Huang and Hong, 2016), dependency (Khanova et al., 2015), critical thinking capability (van Vliet et al., 2015), creativity (Al-Zahrani, 2015), and problem-solving capability (Chen et al., 2015; Akçayir and Akçayir, 2018). The conclusion has been reached that both cooperative learning method and flipped classroom applications fall under the umbrella of active learning as a result of literature review (Bergmann and Sams, 2012, 2016; Tucker, 2012; Foldnes, 2016). Therefore, it is thought that the cooperative learning method will be effective in carrying out the class activities needed by the flipped classroom method.

*Proposition 4:* Cooperative learning method and flipped classroom are among the active learning approaches based on constructivist theory. Utilizing the cooperative learning method in flipped classroom environment will increase the effectiveness of the teaching-learning process compared to individual use of the flipped classroom and the cooperative learning method.

### Cooperative Learning Method in Flipped Classroom Environment

Up to this point in the research, we tried to present the relation between the flipped classroom and the cooperative learning method. At this point, we will make some suggestions on how the cooperative learning method can be integrated into flipped classroom.

The research conducted by Munir et al. (2018) on how to include cooperative learning into the flipped classroom comes as an important beginning. In application of cooperative learning method to a flipped classroom environment, students watch the content prepared by the teacher as a video before the lesson and prepare for the class. When they come to class, a cooperative learning method is applied, and the students carry out learning activities in small groups. Apart from that, teaching activities such as group discussion, teaching basic concepts, and providing feedback are carried out by the teacher within the lesson (Munir et al., 2018). However, this research has only tackled the cooperative learning method as a general approach and has not employed the techniques of the method. Several techniques have been developed on how to apply the cooperative learning method more effectively and openly in the application process. The most used and most researched of these techniques are Learning Together (Johnson et al., 2007), Student Teams and Achievement Divisions (STAD) (Slavin, 1994), Group Research (Thelen, 1981; Sharan and Sharan, 1992), and Jigsaw (Aronson et al., 1978).

#### Learning Together Technique

In this learning together technique, certain roles are given to students, and they are appointed into heterogeneous groups. Students strive to achieve the common group objective in different roles in these groups; that is, each student completes the part of the work he/she is assigned to Johnson and Johnson (2002).

In the learning together technique to be used in the flipped classroom environment, a separate video can be prepared for each role and subject part of each student. Therefore, each student views the video related to his/her issue field at home, and he/she can combine the parts he/she viewed in classroom environment when he/she comes to school. As another method, after each student watches the same video, the activity or homework to be done is divided among students, and each student carries out his/her own learning task.

#### Student Teams and Achievement Divisions

This technique was developed by Slavin and his colleagues in John Hopkins University (Slavin, 1994). Similar to other cooperative learning techniques, students work in small heterogeneous groups together to achieve learning goals and master the subject, and each student is responsible for the learning of his/her group mates in STAD. The main characteristic of the technique is that the team goals and achievement can be achieved only when all the group members achieve their individual goals. Students are given short tests on the subject, and they earn a certificate or rewards as a result of the points they get from this test. There are also team awards in this technique, and these awards motivate the students to help other group members (Slavin, 1994, 2015; Sharan, 2015). According to Slavin (1995), if the group reward is given for only one work, it would not motivate each student to study. In this case, some students will be reluctant to explain the subject to their friend, or one or two students might have to carry out the whole group's work.

In a STAD technique to be used in flipped classroom environment, the students might control whether their classmates have watched the prepared videos. They might also be evaluated by the teacher according to the degree to which they follow the lesson content transmitted out of the school. By this means, each student may be expected to be included in the extracurricular learning process for his/her group's achievement and therefore engage in integration both in his/her group and to his/her subject.

#### Jigsaw

In this technique, the students are assigned to learning groups consisting of six people. One subject is divided into six equal parts, and each student is assigned a part. These parts can be visualized like a jigsaw; when they are combined, they become a full learning product. Each student must learn his/her part well. Thus, he/she can provide for his/her classmates' learning of that part. In this technique, students are the expert in their part of the subject (Aronson et al., 1978).

Jigsaw is one of the most important techniques that may be adapted to flipped classroom. The lesson content to be prepared as a video is prepared separately for each group. Therefore, when the students come to the classroom, they will need the information of other groups for the studies to be carried out. The students will come together with the members of other groups in order to learn the respective part of each group, and they will return to their groups and share what they learned from the expert group with their group classmates.

*Proposition 5:* The cooperative learning method must be applied by using learning techniques such as cooperative learning, STAD, and jigsaw while integrating to a flipped classroom, as opposed to a general approach.

## Conclusion, Discussion, and Suggestions for Future Research

Flipped classroom and cooperative learning methods are teaching methods that support the approaches stated by Vygotsky. It is suggested to utilize cooperative learning method together with flipped classroom, because the conceptual roots of both are based on active learning and constructivist philosophy, and the existing research has concluded that they are effective (Chen et al., 2015; Foldnes, 2016; Guo et al., 2018; Munir et al., 2018; Zhang, 2018).

This research has reviewed the flipped classroom and cooperative learning method in aspect of Vygotsky's learning theories. The research provided five propositions. We can classify these five propositions in the context of transmission of culture, social interaction, ZPD, active learning approaches, and cooperative learning techniques.

Flipped classroom is important as a culture transmission tool, because it uses videos during extracurricular time and supports enculturation of the individual by a more knowledgeable person and therefore ZPD (Steen-Utheim and Foldnes, 2018; Zheng et al., 2020). Also, in social aspect, the social structures, tools, and values are transmitted to students in extracurricular time.

Researchers have expected the conclusion that the flipped classroom supports ZPD to a greater extent (Little, 2015). However, it is suggested to use the cooperative learning method during class time in order to realize this condition (Bishop and Verleger, 2013; Foldnes, 2016; Fox and Docherty, 2019). Therefore, the cooperative learning method will provide for better interaction of students with each other. As a result of this interaction, the more knowledgeable peers will carry out information transmission to their less advanced peers, and the disadvantaged peers will benefit from this. As a result of ZPD, the individual will work in the same group with a more knowledgeable peer and will improve themselves. Also, in a flipped classroom, the videos prepared by the teacher and more knowledgeable peers (Steen-Utheim and Foldnes, 2018; Zheng et al., 2020) will support the individual's learning.

Cooperative learning in flipped classroom method, which is a combination of both methods, supports holistic use of authentic activities, makes the students need social interaction, and realizes the change process of the individual when reviewed from the aspect of Vygotsky theory (Doolittle, 1997). Therefore, utilizing the cooperative learning method together with the flipped classroom method matches perfectly in aspect of constructivism, active learning, and Vygotsky approaches (Bishop and Verleger, 2013; Hayashi et al., 2015; Kanjug et al., 2018; Eryilmaz and Cigdemoglu, 2019). It is also thought to bring great benefit for the students.

Cooperative learning and flipped classroom are among the active learning approaches based on constructivist theory (Felder, 2012; Bishop and Verleger, 2013; Jensen et al., 2015; Foldnes, 2016). Utilizing cooperative learning method in flipped classroom environment will increase the effectiveness of the teaching-learning process compared to individual use of each flipped classroom and the cooperative learning method. When flipped classroom and cooperative learning method are used together, research has shown that they have positive impacts on student success, as well as attitudes and behaviors (Chen et al., 2015; Foldnes, 2016; Guo et al., 2018; Munir et al., 2018; Zhang, 2018).

Cooperative learning method in flipped classroom environment must be planned well, and the cooperative learning techniques must be used within class time. It is stated with time that cooperative learning must be handled as a pedagogic umbrella or an approach, as opposed to a technique or method (Sharan, 2015). It is suggested that the cooperative learning techniques are applied selectively according to different subjects and learning gains (Slavin, 1996; Johnson et al., 2000). Cooperative learning techniques, such as learning together, group investigation, STAD, and Jigsaw, can be adapted to the classroom easily and applied by teachers.

The following suggestions are made as a result of the research conclusion:

- Research is needed in qualitative, quantitative, and mixed research patterns for applications of cooperative learning method in flipped classroom method in all age groups and in different subjects.
- The research to be made must be carried out on mediator variables such as motivation, student attitudes, cohesion, and peer relations that may have a role on impacting academic success as opposed to relying on solely academic success.

## Author Contributions

The author confirms being the sole contributor of this work and has approved it for publication.

## Conflict of Interest

The author declares that the research was conducted in the absence of any commercial or financial relationships that could be construed as a potential conflict of interest.
